# Whole-genome sequences of 38 siderophore-producing isolates from root systems of two pea and one wheat varieties

**DOI:** 10.1128/mra.00570-25

**Published:** 2025-07-23

**Authors:** Liya Zhu, Céline Lopez-Roques, Marine Sallaberry, Kory Jason, Laurent Ouerdane, Sylvie Mazurier, Philippe Lemanceau, Barbara Pivato

**Affiliations:** 1 Agroécologie, INRAE, Institut Agro, Université Bourgogne Europe 27011, Dijon, France; 2 INRAE, GeT-PlaGe, Genotoul 27057 , Castanet-Tolosan, France; 3 Universite de Pau et des Pays de l'Adour, CNRS, IPREM, E2S UPPA 27073, Pau, France; The University of Arizona, Tucson, Arizona, USA

**Keywords:** bacterial isolates, root system, iron, siderophore

## Abstract

Here, we report the whole-genome sequencing of 38 siderophore-producing bacterial strains isolated from the root systems of two pea and one wheat varieties. High-quality genomic data for each isolate was generated using PacBio HiFi sequencing, producing 6.1 Gb of HiFi reads and achieving an average assembly completeness of 98.85%.

## ANNOUNCEMENT

Iron is abundant in soils but mostly unavailable to plants and microorganisms (1). Many bacteria secrete siderophores—iron-chelating secondary metabolites—that facilitate iron acquisition and enhance plant uptake (2–4). Beyond nutrition, siderophores mediate microbial communities' interactions, including cooperation, competition, and exploitation, influencing community assembly, stability, and plant health (5, 6).

Here, we report whole-genome sequencing of 38 siderophore-producing bacterial strains isolated from the root system of two pea (*Pisum sativum* L., Balltrap and Dexter) and one wheat (*Triticum aestivum* L., Flamenko) varieties (7). Plants were grown in two previously characterized soil types (7) under laboratory conditions (47°19′1.268″ N, 5°4′16.075″ E). Roots were rinsed, crushed, and serial dilutions plated on 0.1× Tryptic Soy Agar (TSA, Condalab) supplemented with 200 µg mL^−1^ cycloheximide (Sigma). Plates were incubated at 25°C for 5 days. Colonies were purified by single-colony isolation and screened for siderophore production using CAS medium (8, 9). Positive isolates were coded (Table 1) and stored at −80°C in 0.1× Tryptic Soy Broth (TSB, Condalab) with 12.5% glycerol (Promega), after 3–5 days of liquid growth.

**TABLE 1:** Taxonomic identification, related metadata, HiFi reads statistics, genome assembly statistics (BUSCO with the alphaproteobacteria_odb10 data set and QUAST), and accession numbers for the 38 bacterial whole-genome sequences^*a*^

		Metadata		HiFi reads statistics		Genome assembly statistics							Accession numbers		
Strain Code^*b*^	Taxonomic Affiliation	Plant	Soil type	Read counts	N50 (bp)	BUSCOs Results	# Contigs	Total Length (bp)	N50 (bp)	L50	GC (%)	Coverage (×)	Biosample accession No.	SRA accession No.	Assembly accession No.
**36a20**	*Acidovorax facilis*	Wheat	Auzeville	21,525	14,783	C:99.2%[S:98.4%,D:0.8%],F:0.8%,M:0.0%,n:124	3	5,809,211	5493086	1	64.34	40	SAMN47300670	SRX27953989	GCA_050627005.1
**37c5**	*Acidovorax facilis*	Wheat	Auzeville	42,293	11,357	C:99.2%[S:98.4%,D:0.8%],F:0.8%,M:0.0%,n:124	3	5,806,590	5490465	1	64.34	61	SAMN47300673	SRX27954012	GCA_050626955.1
**21a14**	*Arthrobacter* sp. 21a14	Pea Dexter	Auzeville	25,977	10,022	C:99.2%[S:98.4%,D:0.8%],F:0.8%,M:0.0%,n:124	2	5,652,313	5437079	1	61.55	35	SAMN47300684	SRX27953995	GCA_050627125.1
**20a6**	*Arthrobacter* sp. 20a6	Pea Dexter	Epoisses	16,628	10,117	C:99.2%[S:98.4%,D:0.8%],F:0.8%,M:0.0%,n:124	3	5,655,880	5437081	1	61.55	22	SAMN47300702	SRX27954015	GCA_050710035.1
**25a33**	*Enterobacter kobei*	Pea Dexter	Auzeville	37,318	16,388	C:99.2%[S:99.2%,D:0.0%],F:0.0%,M:0.8%,n:124	2	4,762,226	4623341	1	55.01	94	SAMN47300682	SRX27953993	GCA_050627095.1
**25b15**	*Enterobacter kobei*	Pea Dexter	Auzeville	17,868	16,800	C:99.2%[S:99.2%,D:0.0%],F:0.0%,M:0.8%,n:124	2	4,762,226	4623341	1	55.01	46	SAMN47300685	SRX27953996	GCA_050627105.1
**4a1**	*Enterobacter kobei*	Pea Balltrap	Epoisses	15,809	15,755	C:99.2%[S:99.2%,D:0.0%],F:0.0%,M:0.8%,n:124	2	4,762,221	4623341	1	55.01	40	SAMN47300696	SRX27954008	GCA_050626945.1
**9c6**	*Enterobacter kobei*	Pea Balltrap	Auzeville	27,194	16,335	C:99.2%[S:99.2%,D:0.0%],F:0.0%,M:0.8%,n:124	2	4,762,226	4623341	1	55.01	71	SAMN47300678	SRX27954025	GCA_050626885.1
**34a33**	*Enterobacter ludwigii*	Wheat	Epoisses	12,914	15,383	C:99.2%[S:99.2%,D:0.0%],F:0.0%,M:0.8%,n:124	2	4983446	4881988	1	54.48	30	SAMN47300691	SRX27954003	GCA_050627035.1
**4a6**	*Enterobacter ludwigii*	Pea Balltrap	Epoisses	24,107	16,628	C:99.2%[S:99.2%,D:0.0%],F:0.0%,M:0.8%,n:124	2	4983447	4881989	1	54.48	61	SAMN47300697	SRX27954009	GCA_050626925.1
**4b24**	*Enterobacter ludwigii*	Pea Balltrap	Epoisses	26,176	16,314	C:99.2%[S:99.2%,D:0.0%],F:0.0%,M:0.8%,n:124	2	4983447	4881989	1	54.48	65	SAMN47300698	SRX27954010	GCA_050626905.1
**19b38**	*Enterobacter mori*	Pea Dexter	Epoisses	26,063	7,611	C:99.2%[S:99.2%,D:0.0%],F:0.0%,M:0.8%,n:124	8	4946610	4907588	1	55.49	31	SAMN47300703	SRX27954016	GCA_050710095.1
**25c27**	*Enterobacter mori*	Pea Dexter	Auzeville	26,278	16,097	C:100.0%[S:23.4%,D:76.6%],F:0.0%,M:0.0%,n:124	1	4911960	4911960	1	55.48	66	SAMN47300683	SRX27953994	GCA_050627085.1
**25b30**	*Massilia eburnea*	Pea Dexter	Auzeville	11,420	15,370	C:77.4%[S:77.4%,D:0.0%],F:2.4%,M:20.2%,n:124	36	4594051	163959	9	62.08	29	SAMN47300687	SRX27953998	GCA_050709955.1
**10c5**	*Microbacterium phyllosphaerae*	Pea Balltrap	Auzeville	90,071	14,152	C:98.4%[S:98.4%,D:0.0%],F:0.8%,M:0.8%,n:124	1	3826298	3826298	1	67.97	264	SAMN47300675	SRX27954022	GCA_050628115.1
**8b26**	*Paenibacillus* sp. 8b26	Pea Balltrap	Auzeville	28,221	18,206	C:100.0%[S:100.0%,D:0.0%],F:0.0%,M:0.0%,n:124	1	6129587	6129587	1	46.44	62	SAMN47300680	SRX27953991	GCA_050626875.1
**10c25**	*Paraburkholderia fungorum*	Pea Balltrap	Auzeville	16,678	13,402	C:100.0%[S:97.6%,D:2.4%],F:0.0%,M:0.0%,n:124	6	9355406	4731166	1	61.74	17	SAMN47300676	SRX27954023	GCA_050710135.1
**18a25**	*Paraburkholderia fungorum*	Pea Dexter	Epoisses	24,018	14,819	C:100.0%[S:97.6%,D:2.4%],F:0.0%,M:0.0%,n:124	4	9322411	4729161	1	61.73	28	SAMN47300704	SRX27954017	GCA_050627395.1
**25c37**	*Paraburkholderia fungorum*	Pea Dexter	Auzeville	19,568	13,825	C:100.0%[S:97.6%,D:2.4%],F:0.0%,M:0.0%,n:124	4	9322396	4729159	1	61.73	21	SAMN47300686	SRX27953997	GCA_050627075.1
**36b22**	*Polaromonas aquatica*	Wheat	Auzeville	29,313	8,352	C:100.0%[S:99.2%,D:0.8%],F:0.0%,M:0.0%,n:124	1	4557853	4557853	1	61.59	41	SAMN47300672	SRX27954001	GCA_050626995.1
**4a21**	*Pseudomonas alvandae*	Pea Balltrap	Epoisses	15,580	14,932	C:100.0%[S:100.0%,D:0.0%],F:0.0%,M:0.0%,n:124	1	6213704	6213704	1	60.96	27	SAMN47300699	SRX27954011	GCA_050626915.1
**18b10**	*Pseudomonas brassicacearum*	Pea Dexter	Epoisses	30,999	9,515	C:100.0%[S:100.0%,D:0.0%],F:0.0%,M:0.0%,n:124	1	6739771	6739771	1	60.87	33	SAMN47300705	SRX27954018	GCA_050627145.1
**22c22**	*Pseudomonas brassicacearum*	Pea Dexter	Auzeville	24,495	9,452	C:100.0%[S:100.0%,D:0.0%],F:0.0%,M:0.0%,n:124	2	6825820	6816008	1	60.81	25	SAMN47300688	SRX27953999	GCA_050710055.1
**35a7**	*Pseudomonas gorinensis*	Wheat	Epoisses	73,210	13,958	C:99.2%[S:99.2%,D:0.0%],F:0.8%,M:0.0%,n:124	2	7145029	7138193	1	60.51	114	SAMN47300692	SRX27954004	GCA_050709935.1
**21c7**	*Pseudomonas gorinensis*	Pea Dexter	Auzeville	96,544	13,640	C:99.2%[S:98.4%,D:0.8%],F:0.8%,M:0.0%,n:124	1	7207491	7207491	1	60.48	142	SAMN47300689	SRX27954000	GCA_050627115.1
**34a15**	*Pseudomonas gorinensis*	Wheat	Epoisses	6,711	19,431	C:99.2%[S:98.4%,D:0.8%],F:0.8%,M:0.0%,n:124	1	7206726	7206726	1	60.48	14	SAMN47300693	SRX27954005	GCA_050627045.1
**9b17**	*Pseudomonas simiae*	Pea Balltrap	Auzeville	36,437	8,757	C:100.0%[S:100.0%,D:0.0%],F:0.0%,M:0.0%,n:124	2	6324885	6316763	1	60.28	39	SAMN47300677	SRX27954024	GCA_050709815.1
**3c1**	*Pseudomonas siliginis*	Pea Balltrap	Epoisses	113,858	13,435	C:100.0%[S:100.0%,D:0.0%],F:0.0%,M:0.0%,n:124	1	5970447	5970447	1	60.04	195	SAMN47300700	SRX27954013	GCA_050626965.1
**25b28**	*Pseudomonas solani*	Pea Dexter	Auzeville	42,504	10,604	C:99.2%[S:99.2%,D:0.0%],F:0.8%,M:0.0%,n:124	3	7144013	3964255	1	65.89	48	SAMN47300690	SRX27954002	GCA_050709975.1
**35c15**	*Pseudomonas thivervalensis*	Wheat	Epoisses	11,620	16,943	C:100.0%[S:100.0%,D:0.0%],F:0.0%,M:0.0%,n:124	1	6495419	6495419	1	61.22	22	SAMN47300694	SRX27954006	GCA_050627015.1
**8a22**	*Pseudomonas thivervalensis*	Pea Balltrap	Auzeville	9,214	12,166	C:99.2%[S:99.2%,D:0.0%],F:0.0%,M:0.8%,n:124	2	6979370	5539090	1	61.01	12	SAMN47300679	SRX27954026	GCA_050709855.1
**4a18**	*Ralstonia holmesii*	Pea Balltrap	Epoisses	5,942	14,080	C:98.4%[S:78.2%,D:3.2%],F:4.0%,M:14.6%,n:124	4	5578591	3593299	1	63.58	12	SAMN47300701	SRX27954014	GCA_050626935.1
**18a19**	*Ralstonia insidiosa*	Pea Dexter	Epoisses	9,068	14,820	C:100.0%[S:99.2%,D:0.8%],F:0.0%,M:0.0%,n:124	4	5999436	3744346	1	63.57	16	SAMN47300707	SRX27954020	GCA_050627405.1
**18c12**	*Ralstonia insidiosa*	Pea Dexter	Epoisses	9,113	14,582	C:100.0%[S:99.2%,D:0.8%],F:0.0%,M:0.0%,n:124	4	6739771	6739771	1	60.87	16	SAMN47300706	SRX27954019	GCA_050627135.1
**37a14**	*Ralstonia thomasii*	Wheat	Auzeville	10,129	15,860	C:99.2%[S:98.4%,D:0.8%],F:0.8%,M:0.0%,n:124	3	5138688	3497269	1	63.80	23	SAMN47300674	SRX27954021	GCA_050626975.1
**37a16**	*Roseateles noduli*	Wheat	Auzeville	11,881	14,537	C:98.2%[S:24.2%,D:0.0%],F:5.6%,M:70.2%,n:124	1	6630955	6630955	1	68.53	20	SAMN47300671	SRX27953990	GCA_050626985.1
**35b25**	*Variovorax paradoxus* NBRC 15149	Wheat	Epoisses	53,373	11,279	C:100.0%[S:100.0%,D:0.0%],F:0.0%,M:0.0%,n:124	2	6043317	5556705	1	64.82	76	SAMN47300695	SRX27954007	GCA_050627025.1
**8a8**	*Variovorax paradoxus* NBRC 15149	Pea Balltrap	Auzeville	7,620	16,223	C:98.4%[S:98.4%,D:0.0%],F:0.8%,M:0.8%,n:124	2	6299093	5159596	1	66.30	15	SAMN47300681	SRX27953992	GCA_050626895.1

^*a*^ BUSCO abbreviations: C = Complete, S = Single copy, D = Duplicated, F = Fragmented, M = Missing; *n* = Number of BUSCO groups searched. Strains are listed in alphabetical order based on their taxonomic affiliation.

^*b*^ Boldface indicates the strain codes in the first column, which are internal laboratory identifiers assigned to each isolate and used throughout the study for traceability purposes.

For DNA extraction, strains were recultivated in 0.1 × TSB and harvested at the end of exponential phase. Genomic DNA was extracted using Genomic-tip 20 /G Kit (Qiagen), quantified with Qubit dsDNA HS (ThermoFisher), and quality-checked by Nanodrop (ThermoFisher) and Agilent Fragment Analyzer (DNA 50 kb kit) (TECAN Infinite 200 Pro). Two µg of DNA per sample were purified and sheared to ~10 kb, using either FastPrep96 (MP Biomedicals) or Megaruptor 3 (Diagenode); no size selection followed shearing.

Libraries were prepared from 500  ng sheared DNA using the SMRTbell Express Template Prep Kit 2.0 (PacBio), ligated with barcoded blunt-end hairpin adapters, treated with exonucleases, and pooled equimolarly.

Sequencing was performed on the PacBio Sequel II system using the Binding Kit 3.2 and Sequencing Kit 2.0, with 100 pM loading concentration, two-hours pre-extension, and 30 hours movie per SMRT cell (3 cells total), using Sequel II DNA Polymerase 2.2.

Subreads were processed using SMRT Link to generate Circular Consensus Sequences (CCS, or HiFi reads), with automatic adapter removal and Q ≥ 20 filtering, generating 6.1 Gb of HiFi reads. Read counts and N50 values are in Table 1.

HiFi reads were assembled with Flye v. 2.9.5 (10). Assembly quality was assessed with BUSCO v5.7.1 (11) with alphaproteobacteria_odb10 data set (11) and QUAST v5.2.0 (12), showing an average completeness of 98.85% (range: 77.40% - 100.00%) and average genome size of 6,067,693 bp (range: 3,826,298 bp - 9,355,406 bp). Chromosomes and plasmids were identified using BLAST v2.16.0. Single-contig assemblies were confirmed circular by Flye reports and submitted as complete genomes to NCBI. Taxonomic classification used Kraken2 v2.1.2 (13) and GTDB-Tk v2.4.0 (14). Assembly summaries are in Table 1.

Genome mining with antiSMASH v7.0 (15) identified 203 biosynthetic gene clusters across the 38 strains, linked to 51 putative secondary metabolites. Sixteen distinct siderophore compounds were annotated via the SIDERITE database (16), including alcaligin, enterobactin, aerobactin, pyoverdine, pyochelin, cepaciachelin, staphyloferrin B, malleobactin, variochelin, desferrioxamine, corrugatin, turnerbactin, methanobactin, histicorrugatin, delftibactin, and pacifibactin.

## Data Availability

The complete genome sequence of the 38 siderophore-producing strains have been submitted to NCBI with bioproject ID PRJNA1234539. The scripts used for the assembly quality assessment are available at https://forgemia.inra.fr/Liya_Zhu/effort_38strains.git.

## References

[1] Robin A, Vansuyt G, Hinsinger P, Meyer JM, Briat JF, Lemanceau P. 2008. Chapter 4 Iron dynamics in the rhizosphere, p 183–225. In Advances in agronomy. Elsevier.

[2] Lurthy T, Cantat C, Jeudy C, Declerck P, Gallardo K, Barraud C, Leroy F, Ourry A, Lemanceau P, Salon C, Mazurier S. 2020. Impact of bacterial siderophores on iron status and ionome in pea. Front Plant Sci 11:730. doi:10.3389/fpls.2020.00730 32595663 PMC7304161

[3] Shirley M, Avoscan L, Bernaud E, Vansuyt G, Lemanceau P. 2011. Comparison of iron acquisition from Fe–pyoverdine by strategy I and strategy II plants. Botany 89:731–735. doi:10.1139/b11-054

[4] Vansuyt G, Robin A, Briat J-F, Curie C, Lemanceau P. 2007. Iron acquisition from Fe-pyoverdine by Arabidopsis thaliana. Mol Plant Microbe Interact 20:441–447. doi:10.1094/MPMI-20-4-0441 17427814

[5] Kramer J, Özkaya Ö, Kümmerli R. 2020. Bacterial siderophores in community and host interactions. Nat Rev Microbiol 18:152–163. doi:10.1038/s41579-019-0284-4 31748738 PMC7116523

[6] Lemanceau P, Expert D, Gaymard F, Bakker P, Briat J-F. 2009. Chapter 12 Role of iron in plant–microbe interactions, p 491–549. In Advances in botanical research. Academic Press.

[7] Semblat A, Turanoglu C, Faivre-Primot C, Lemaître J-P, Marchand D, Dufayet V, Rouet P, Avoscan L, Mazurier S, Lemanceau P, Journet E-P, Pivato B. 2025. Impact of pea-wheat intercropping on grain ionome in relation with changes in Pseudomonas spp. and Enterobacterales abundances. Plant Soil 509:261–288. doi:10.1007/s11104-024-06861-x

[8] Louden BC, Haarmann D, Lynne AM. 2011. Use of blue agar CAS assay for siderophore detection. J Microbiol Biol Educ 12:51–53. doi:10.1128/jmbe.v12i1.249 23653742 PMC3577196

[9] Arora NK, Verma M. 2017. Modified microplate method for rapid and efficient estimation of siderophore produced by bacteria. 3 Biotech 7:381. doi:10.1007/s13205-017-1008-y PMC5658296 29109926

[10] Kolmogorov M, Yuan J, Lin Y, Pevzner PA. 2019. Assembly of long, error-prone reads using repeat graphs. Nat Biotechnol 37:540–546. doi:10.1038/s41587-019-0072-8 30936562

[11] Manni M, Berkeley MR, Seppey M, Simão FA, Zdobnov EM. 2021. BUSCO update: novel and streamlined workflows along with broader and deeper phylogenetic coverage for scoring of eukaryotic, prokaryotic, and viral genomes. Mol Biol Evol 38:4647–4654. doi:10.1093/molbev/msab199 34320186 PMC8476166

[12] Gurevich A, Saveliev V, Vyahhi N, Tesler G. 2013. QUAST: quality assessment tool for genome assemblies. Bioinformatics 29:1072–1075. doi:10.1093/bioinformatics/btt086 23422339 PMC3624806

[13] Wood DE, Lu J, Langmead B. 2019. Improved metagenomic analysis with Kraken 2. Genome Biol 20:257. doi:10.1186/s13059-019-1891-0 31779668 PMC6883579

[14] Chaumeil P-A, Mussig AJ, Hugenholtz P, Parks DH. 2020. GTDB-Tk: a toolkit to classify genomes with the genome taxonomy database. Bioinformatics 36:1925–1927. doi:10.1093/bioinformatics/btz848 PMC7703759 31730192

[15] Blin K, Shaw S, Augustijn HE, Reitz ZL, Biermann F, Alanjary M, Fetter A, Terlouw BR, Metcalf WW, Helfrich EJN, van Wezel GP, Medema MH, Weber T. 2023. antiSMASH 7.0: new and improved predictions for detection, regulation, chemical structures and visualisation. Nucleic Acids Res 51:W46–W50. doi:10.1093/nar/gkad344 37140036 PMC10320115

[16] He R, Gu S, Xu J, Li X, Chen H, Shao Z, Wang F, Shao J, Yin W-B, Qian L, Wei Z, Li Z. 2024. SIDERITE: Unveiling hidden siderophore diversity in the chemical space through digital exploration. iMeta 3:e192. doi:10.1002/imt2.192 38882500 PMC11170966

